# Preparation of β(1→3)/β(1→4) xylooligosaccharides from red alga dulse by two xylanases from *Streptomyces thermogriseus*

**DOI:** 10.1186/s40643-021-00390-6

**Published:** 2021-05-12

**Authors:** Yuki Fujii, Manami Kobayashi, Yoshikatsu Miyabe, Hideki Kishimura, Tadashi Hatanaka, Yuya Kumagai

**Affiliations:** 1 Chair of Marine Chemical Resource Development, Graduate School of Fisheries Sciences, Hokkaido University, Hakodate, Hokkaido 041-8611 Japan; 2 Aomori Prefectural Industrial Technology Research Center, Food Research Institute, 221-10 Yamaguchi, Nogi, Aomori, Aomori-ken 030-0142 Japan; 3 Laboratory of Marine Chemical Resource Development, Faculty of Fisheries Sciences, Hokkaido University, Hakodate, Hokkaido 041-8611 Japan; 4 Okayama Prefectural Technology Center for Agriculture, Forestry, and Fisheries, Research Institute for Biological Sciences (RIBS), 7549-1 Kibichuo-cho, Kaga-gun, Okayama, 716-1241 Japan

**Keywords:** *Palmaria palmata* in Japan, β(1→3)/β(1→4)-xylooligosaccharides production, GH10 and GH11 endoxylanase, Actinomycete

## Abstract

Red alga dulse contains xylan with β(1→3)/β(1→4) linkages. We previously prepared xylooligosaccharides (XOSs) from dulse xylan; however, the product contained many d-xylose residues and fewer XOSs with β(1→3) linkages. To improve the efficiency of XOS production, we prepared two recombinant endoxylanases from *Streptomyces thermogriseus* (StXyl10 and StXyl11). Comparing the *k*_cat_/*K*_m_ values for dulse xylan, this value from StXyl10 was approximately two times higher than that from StXyl11. We then determined the suitable conditions for XOS production. As a result, dulse XOS was prepared by the successive hydrolysis of 10 mg/mL dulse xylan by 0.5 μg/mL StXyl10 for 4 h at 50 °C and then 2.0 μg/mL StXyl11 for 36 h at 60 °C. Xylan was converted into 95.8% XOS, including 59.7% XOS with a β(1→3) linkage and 0.97% d-xylose. Our study provides useful information for the production of XOSs with β(1→3) linkages.

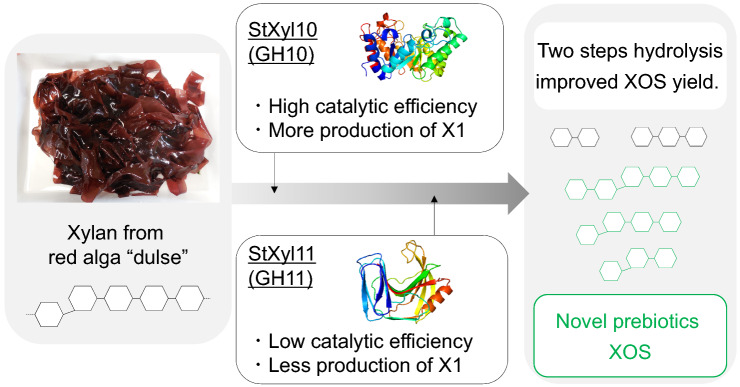

## Introduction

Xylan is very useful in the production of biofuels, pulp, fiber and foods (Buchert et al. 1994; Dodd and Cann 2009; Duarte et al. 2012). In the food industry, xylan is converted to d-xylose (X1) or xylooligosaccharides (XOSs). X1 is used as a low calorie sweetener, a source for xylitol and a coloring agent via the Maillard reaction (Akpinar et al. 2009). XOSs are stable at low pH and high temperatures up to 100 °C (Carvalho et al. 2013; Singh et al. 2015). XOSs function by decreasing blood sugar, lipids and oxidative status in type two diabetes mellitus (Gobinath et al. 2010; Sheu et al. 2008). In addition, XOSs are known as prebiotics, showing various beneficial effects on human health (Okazaki et al. 1990; Zhu et al. 2015).

Land plant cell walls are complex structures consisting of cellulose, hemicellulose, pectin and lignin. The main hemicelluloses are xylan and mannan. Land plant xylan forms a xylan–cellulose–lignin complex by covalent and noncovalent bonds (Akpinar et al. 2009; Zhu et al. 2006; Guillaume et al. 2019), and the formed structure resists enzymatic hydrolysis. Therefore, pretreatment with acid or alkali is necessary for the enzymatic hydrolysis of xylan complexes (Carvalho et al. 2013; de Figueiredo et al. 2017; Lyu et al. 2018; Rajagopalan et al. 2017). Red algae also possess cell walls without lignin and with less cellulose (Martone et al. 2009). Although the main polysaccharides of red algae are usually galactan, agar and carrageenan, some red algae, such as Nemaliophycidae, possess xylan in their cell wall (Chen et al. 1986; Nerinckx et al. 2004). The structure of red algal xylan mainly consists of one β(1→3) linkage for every β(1→4) xylotetraose (Deniaud et al. 2003; Nerinckx et al. 2004; Viana et al. 2011). Because of the mixed-linked structure, the interaction between xylans is loose (Lahaye et al. 2003), indicating that red algal xylan has advantages in the enzymatic production of XOSs. Red alga dulse contains xylan with minor amounts of cellulose and β-(1→4)-xylan in the cell wall (Morgan et al. 1980). The major components of dulse are proteins (approximately 40 g/100 g dried dulse) (Furuta et al. 2016; Miyabe et al. 2017). Therefore, we have studied these components and their functions on human health, such as their antioxidant activity and inhibition of angiotensin I-converting enzyme activity (Kumagai et al. 2019a, b; Nishida et al. 2020; Sato et al. 2019). Xylan is the second main ingredient of dulse. We attempted to prepare XOSs from dulse xylan (DX) using commercial enzymes (Yamamoto et al. 2019). Then, we purified β(1→3) xylosyl xylobiose (DX3) and clarified their action as prebiotics on *Bifidobacterium* sp. (Kobayashi et al. 2020).

Endo-1,4-*β*-xylanase (EC 3.2.1.8) is one of the main enzymes for xylan hydrolysis (Chapla et al. 2012). These types of enzymes are mainly classified into glycoside hydrolase (GH, http://www.cazy.org/) families 10 and 11 (Álvarez-Cervantes et al. 2016; Paës et al. 2012; Yagi et al. 2019). GH10 folds into a (β/α)_8_ barrel structure and hydrolyzes xylans that have a side chain (Biely et al. 1997). The hydrolysis products, X1 and XOS, have a small degree of polymerization (DP) (Meng et al. 2015; Rahmani et al. 2019). GH11 folds into a *β*-jerry roll structure and also hydrolyzes xylans with side chains. The hydrolysis products are XOSs with a large DP and less X1. The differences in hydrolysis products come from the structures of the enzymes. GH10 xylanases usually possess five to seven subsites. These subsites incorporate X1 with α(1→3)-arabinofuranose and α(1→2)-4-*O*-methylglucuronic acid side chains, except for subsite 1 (Fujimoto et al. 2004). The subsites of GH11 xylanases usually possess subsites − 2 to + 3, and subsites − 2 to + 1 do not incorporate X1 with side chains (Vardakou et al. 2008). The use of xylanases with different substrate specificities leads to the efficient production of XOSs with β(1→3) linkages.

In this study, we prepared and characterized a GH10 and GH11 endoxylanase from *Streptomyces thermogriseus* (StXyl10 and StXyl11, respectively). Next, we attempted to produce XOSs with less X1 and more β (1→3) linkages from DX by using these two xylanases.

## Results and discussion

### Cloning and sequence analysis of xylanases from *S. thermogriseus*

Sequencing of the genomic DNA of *S*. *thermogriseus* NBRC100772 was performed using a PacBio next-generation sequencer, and their annotations were performed with the Prokka program. Using PCR methods, two *S*. *thermogriseus* xylanase genes (stxyl10 and stxyl11) were cloned. Consequently, we determined nucleotide sequences of 1434 bp for stxyl10 encoding an amino acid sequence of 477 residues, and 996 bp for stxyl11 encoding an amino acid (AA) sequence of 331 residues. The signal peptide for secretion was predicted by the SignalP 4.1 server (http://www.cbs.dtu.dk/services/SignalP-4.1/). StXyl10 consists of AA residues 1–41 as the signal peptide and AA residues 42–477 for the mature protein composed of the GH10 catalytic domain, linker and carbohydrate-binding module (CBM) 13. StXyl11 consisted of AA residues 1–41 as the signal peptide and AA residues 42–331 for the mature protein composed of the GH11 catalytic domain, linker and CBM2. StXyl10 showed 99.8% identity with GH10 *Streptomyces thermovulgaris* TISTR1948 xylanase (NCBI accession number (AN): LC088500) and XynST10 from *Streptomyces* sp. B6 (AN: MN420656) (Boonchuay et al. 2016; Chaiyaso et al. 2011; Liu et al. 2020). Comparison of CBM2 with StXyl10 showed the substitution of Ile475 with Thr475. The primary structure of StXyl11 showed 100% identity with GH11 from *Streptomyces* sp. B6 XynST11 (AN: MN420657) and 99.3% identity with an endoxylanase from *Streptomyces thermonitrificans* NTU-88 (AN: ABF72145), which is named *S*. *thermovulgaris* in the NCBI (Cheng et al. 2008).

### Biochemical characterization of the xylanases

**Fig. 1:**
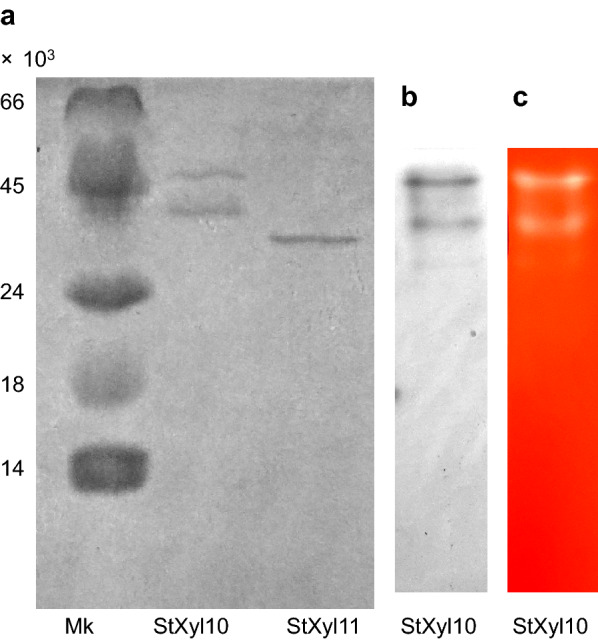
SDS-PAGE and native-PAGE of purified xylanases. **a** SDS-PAGE was performed on a 15% acrylamide gel, and 1 μg of the purified enzymes was applied. **b** CBB staining of native-PAGE of 2 μg of purified StXyl10. **c** Congo red staining of agar plates containing 0.5% GX hybridized with native-PAGE

StXyl10 and StXyl11 were expressed in *Escherichia coli*. The purities of xylanases were evaluated by SDS-PAGE (Fig. 1a). StXyl10 showed two bands with molecular weights of 38,600 and 46,900. The deduced molecular weight of StXyl10 with a 6 × His-tag was 46,990, which corresponded to the larger band. We therefore speculated that the larger band was mature StXyl10. The expressed enzymes in this study possessed a 6 × His-tag at the N-terminus. The small band was estimated to be the loss of approximately 30 AAs from the C-terminus. CBM13 consists of approximately 150 AA. It has been reported that the N-terminus of CBM13 is required for substrate binding (PDB: 1V6X) (Fujimoto et al. 2004). In addition, the small band was not found in the purified StXyl11, meaning that this was not due to bacterial contamination. To confirm the small band of StXyl10, we performed zymogram analysis (Fig. 1b, c). Two active bands, which corresponded to CBB staining densities, were found from the zymography analysis, and we considered these two bands as StXyl10. StXyl11 showed a single band at a MW of 32,400 MW, which was the same as the deduced molecular weight of 32,409 containing a 6 × His-tag.

**Fig. 2:**
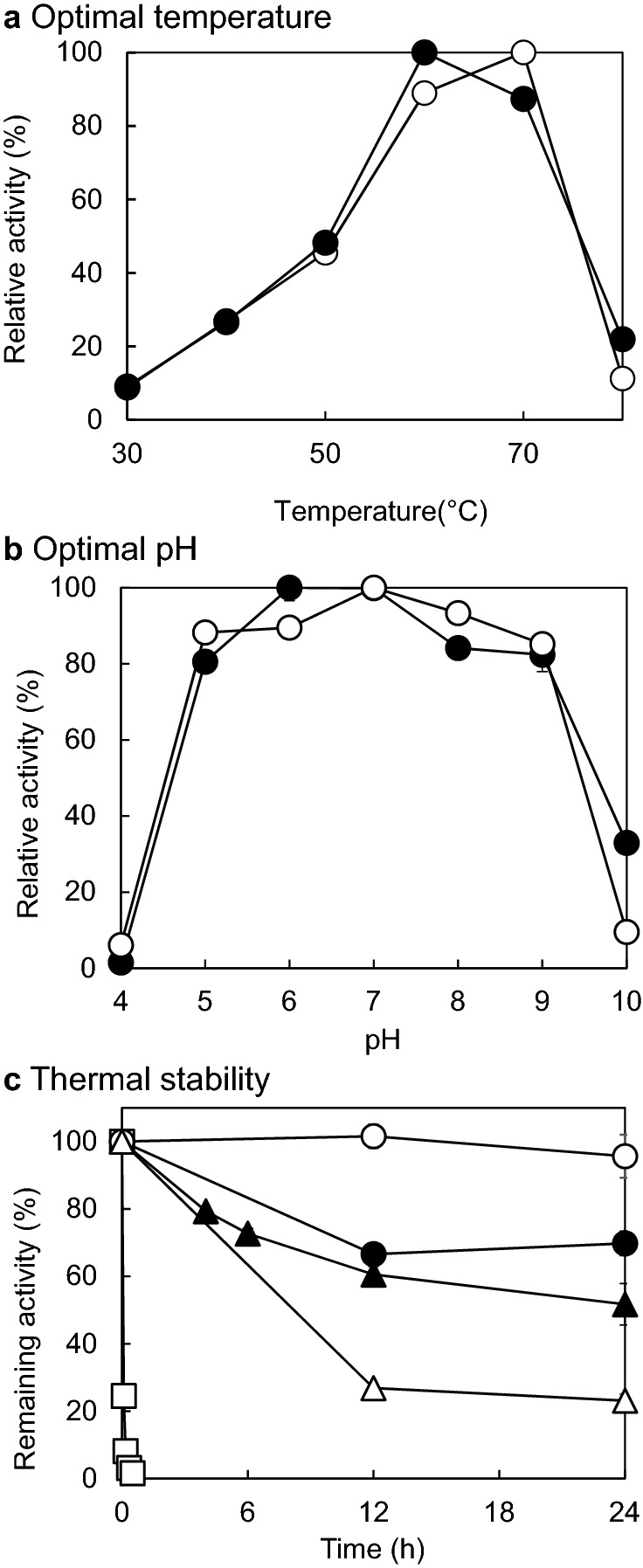
Optimum temperature and pH and thermostability of StXyl10 and StXyl11. **a** Optimum temperatures of StXyl10 and StXyl11. The activities were measured at pH 6.5 and at 30–80 °C in a reaction mixture containing 10 mg/mL GX. White filled circle StXyl10; Black filled circle StXyl11. **b **Optimum pH of StXyl10 and StXyl11. The activities were measured at 65 °C in the reaction mixtures adjusted to pH 4.0–10.0. White filled circle StXyl10; Black filled circle StXyl11. **c** Thermal stabilities of StXyl10 and StXyl11. The remaining activities were assessed after incubation of each enzyme at 50–70 °C for up to 24 h in 10 mM sodium phosphate buffer (pH 6.5). Average values for triplicate measurements are shown with standard deviations, and error bars are within the symbols. The standard deviation within the symbols was less than 5% in all cases. White filled circle StXyl10 at 50 °C; White filled triangle StXyl10 at 60 °C; White filled square StXyl10 at 70 °C; Black filled circle StXyl11 at 50 °C; Black filled traingle StXyl11 at 60 °C. The specific activities of StXyl10 and StXyl11 for GX were 252 U/mg at 70 °C and pH 6.5 and 1392 U/mg at 60 °C and pH 6.5, respectively

The optimal temperatures of StXyl10 and StXyl11 were 70 °C and 60 °C, respectively (Fig. 2a). The optimal pH ranges of StXyl10 and StXyl11 were pH 5.0–8.0 and pH 6.0–7.0, respectively (Fig. 2b). StXyl10 was stable at 50 °C for 24 h. The thermal stability of StXyl10 at 60 °C decreased by 27% over 12 h. The half inactivation of StXyl10 at 70 °C was 3.3 min. The thermal stabilities of StXyl11 at 50 °C and 60 °C over 24 h were 70% and 52%, respectively (Fig. 2c). Under the optimal conditions, StXyl10 and StXyl11 showed specific activities of 252 U/mg at 70 °C and 1392 U/mg at 60 °C, respectively. These enzymatic characteristics were similar to those of mesophilic *Streptomyces* xylanases (Boonchuay et al. 2016; Chaiyaso et al. 2011; Cheng et al. 2008; Liu et al. 2020).

The kinetic parameters of StXyl10 for glucuronoxylan (GX) and DX were 675 and 858/s (mg/mL)^−1^, respectively. The StXyl11 values for GX and DX were 610 and 445/s (mg/mL)^−1^, respectively (Table 1). Comparing the DX parameters of StXyl10 and StXyl11, the *k*_cat_/*K*_m_ value of StXyl10 was approximately two times higher. This is the first report that measured the kinetic parameters of DX.

**Table 1:** Kinetic parameters of StXyl10 and StXyl11

Enzyme	Substrate	*K*_m_ mg/mL	*V*_max_ μmol/(mg/min)	*k*_cat_ s^−1^	*k*_cat_/*K*_m_ s^−1^ (mg/mL)^−1^
StXyl10	GX	3.42	2950	2300	675
	DX	3.52	3590	3020	858
StXyl11	GX	14.2	16,300	8690	610
	DX	12.0	10,200	5340	445

### Substrate specificities of the xylanases

**Fig. 3:**
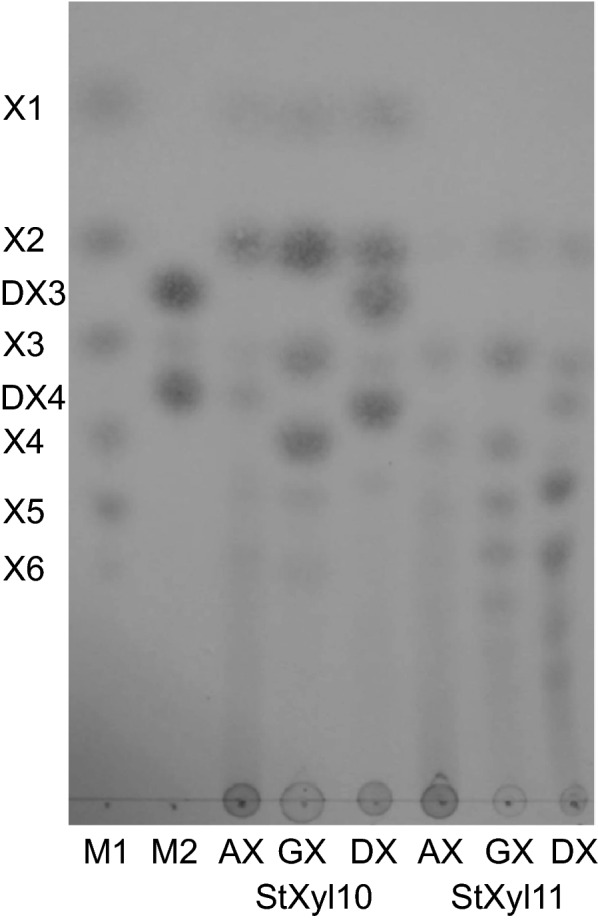
TLC analyses for the hydrolysis products of xylan by StXyl10 and StXyl11. Ten milligrams per milliliter xylan (AX, DX and GX) in 10 mM sodium phosphate buffer (pH 6.5) was hydrolyzed with 1.0 μg/mL StXyl10 at 50 °C for 24 h and 1.0 μg/mL StXyl11 at 60 °C for 24 h. One microliter of each mixture was applied for TLC analysis. M1, marker of X1-X6; M2, marker of DX3 and DX4; AX, arabinoxylan; DX, dulse xylan; GX glucuronoxylan

The hydrolysis products of arabinoxylan (AX), GX and DX by StXyl10 and StXyl11 were evaluated by TLC (Fig. 3). The hydrolysis products of StXyl10 were composed of X1 and XOSs having a short DP, xylobiose (X2), and xylotetraose having one β(1→3) xylosyl linkage (DX4). Imaging analysis showed that the ratios of the main hydrolysis products of DX were as follows: X2, 23.2%; DX3, 25.2%; and DX4, 27.6%. On the other hand, the main hydrolysis products of StXyl11 showed a larger content of XOSs than X4. The hydrolysis products of StXyl11 did not contain X1 from the three substrates or DX3 from DX. From these results, StXyl10 produced a shorter DP than StXyl11 among the three substrates.

### Hydrolysis products by using the xylanases

**Fig. 4:**
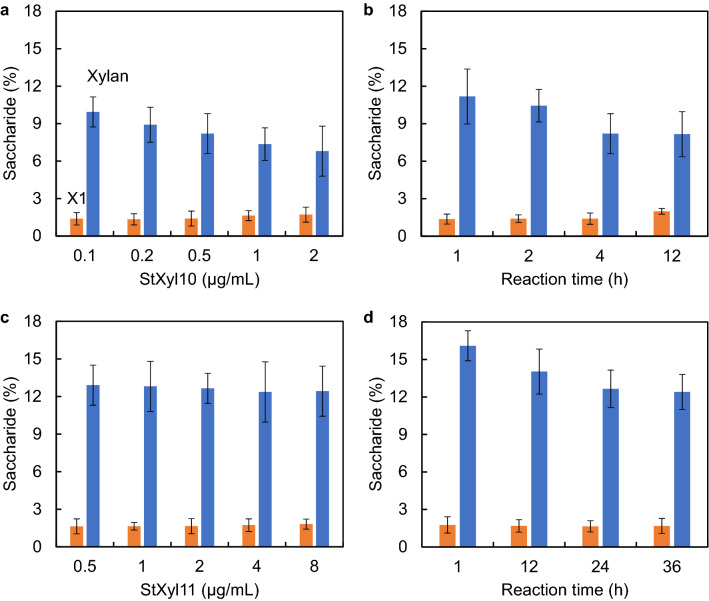
Hydrolysis of DX by StXyl10 and StXyl11. The hydrolysis products were developed by TLC, and the densities of X1 and DX were evaluated by ImageJ. The amounts of X1 and DX under hydrolysis conditions (0.5 μg/mL StXyl10 for 4 h and 2.0 μg/mL StXyl11 for 24 h) were determined by a d-xylose analysis kit and gel filtration, respectively. The amounts of X1 and DX under the other conditions were obtained by TLC densities. **a** DX was hydrolyzed by 0.1–2.0 μg/mL StXyl10 for 4 h at 50 °C and pH 6.5. **b** DX was hydrolyzed by 0.5 μg/mL StXyl10 for 1–12 h at 50 °C and pH 6.5. **c** DX was hydrolyzed by 0.5–8.0 μg/mL StXyl11 for 24 h at 60 °C and pH 6.5. **d** DX was hydrolyzed by 2.0 μg/mL StXyl11 for 1–36 h at 60 °C and pH 6.5

For the efficient production of XOSs from DX without the production of X1, the hydrolysates from StXyl10 and StXyl11 were evaluated (Fig. 4). We first determined the reaction conditions of enzyme concentration and reaction time for each enzyme. To evaluate the enzyme concentrations, DX was hydrolyzed for 4 h at concentrations of 0.1–2.0 μg/mL for StXyl10 and for 24 h at concentrations of 0.5–8.0 μg/mL for StXyl11. To evaluate the reaction times, DX was hydrolyzed at 0.5 μg/mL StXyl10 for 1–12 h and 2.0 μg/mL StXyl11 for 1–36 h. The hydrolysis products were separated by TLC, and the amounts of X1 and DX were evaluated by the band intensities.

When DX was hydrolyzed for 4 h with StXyl10 at the concentrations of 0.1–2.0 μg/mL, a relationship between a decrease in DX and an increase in X1 was found (Fig. 4a), except for the concentration of 0.1 μg/mL StXyl10. This concentration of StXyl10 produced large DP substrates at the origin of the TLC plate. The increase in X1 exceeded the decrease in DX in the hydrolysate by 1.0 μg/mL StXyl10. Therefore, we selected a concentration of 0.5 μg/mL StXyl10 to evaluate the reaction time. The reaction time was evaluated from 1 to 24 h (Fig. 4b). Although the amount of X1 was stable up to 4 h, the amount of X1 increased from 4 to 12 h without decreasing the amount of DX in the hydrolysate. From these results, we determined the DX hydrolysis conditions to be 0.5 μg/mL StXyl10 for 4 h.

Then, the DX hydrolysis conditions with StXyl11 were evaluated. When DX was hydrolyzed for 24 h at StXyl11 concentrations of 0.5–8.0 μg/mL, the decrease in DX was faster than the increase in X1 at concentrations of StXyl11 up to 2.0 μg/mL (Fig. 4c). The decrease in DX was slower than the increase in X1 from 2.0 to 8.0 μg/mL StXyl11. Therefore, we selected a concentration of 2.0 μg/mL StXyl11 to evaluate the reaction time. The reaction time was evaluated from 1 to 36 h. Although the amount of X1 was low at the tested times, the amount of DX decreased up to 24 h and remained almost unchanged for 36 h.

**Fig. 5:**
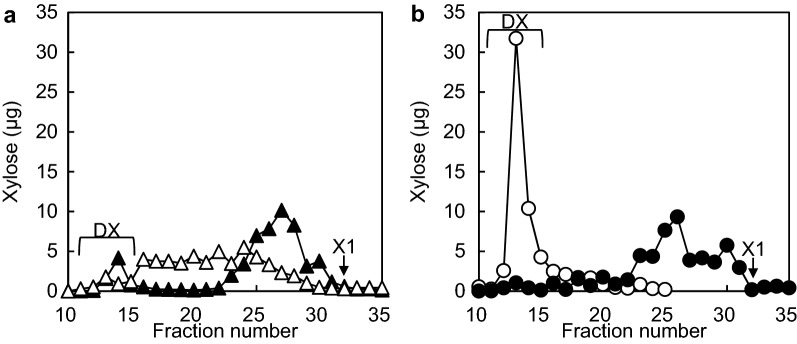
Gel filtration of DX and the hydrolysates from StXyl10, StXyl11 and their combination. The hydrolysates were separated with a Superdex peptide 10/300 GL column. The amounts of each sugar were detected by the phenol-sulfate method. **a** White filled circle DX; Black filled circle StXyl10 and StXyl11; **b** White filled triangle StXyl10; White filled square StXyl11

The hydrolysates under the above conditions were confirmed by gel filtration. The hydrolysate of StXyl10 gave a larger DP (fractions 16–22). On the other hand, the hydrolysate of StXyl11 contained XOSs, but unhydrolyzed DX still remained (Fig. 5a). The amount of intact DX was 8.2% in the StXyl10 hydrolysate and 12.4% in the StXyl11 hydrolysate. When the reaction time of StXyl10 increased, the amount of X1 increased. Even though the reaction time and dose of StXyl11 increased, unhydrolyzed DX remained. We therefore evaluated the successive hydrolysis of DX by both StXyl10 and StXyl11.

### Successive hydrolyses of DX by the two xylanases

For the successive hydrolysis of DX, the hydrolysis conditions of StXyl10, which were determined in the previous Section “Hydrolysis products by using the xylanases” (0.5 μg/mL StXyl10 for 4 h), were employed for limited degradation. After the denaturation of StXyl10 at 100 °C for 10 min, a second hydrolysis by StXyl11 was evaluated (Table 2). Although the amount of X1 did not increase under the tested conditions, the amount of DX decreased in the sample after 36 h of hydrolysis (Table 2). The sample prepared with 2.0 μg/mL StXyl11 for 36 h, contained less DX in the tested successive hydrolysis conditions than that from single hydrolysis. Therefore, we determined the successive hydrolysis conditions as follows: 0.5 μg/mL StXyl10 for 4 h and then 2.0 μg/mL StXyl11 for 36 h. The composition of the hydrolysis products was evaluated in the next section.

**Table 2:** Hydrolysis of DX by the successive reaction with StXyl10 and StXyl11

Enzyme/product	Hydrolysis conditions/relative value				
StXyl11	None^a^	0.5 μg/mL		2.0 μg/mL	
		24 h	36 h	24 h	36 h^b^
X1	1.056	1.011	0.983	1.002	1.000
DX	1.918	1.580	1.077	1.131	1.000

Relative values were obtained by the densities on TLC

Hydrolysis by StXyl11 was performed in 10 mM sodium phosphate (pH 6.5) at 60 °C

^a^The product was treated with 0.5 μg/mL StXyl10 at pH 6.5 and 50 °C for 4 h

^b^An X1 and DX of 1.000 correspond to 0.94% and 3.3%, respectively

### Evaluation of the hydrolysis products by the xylanase combination hydrolysis

The hydrolysis ratio of the sample was determined by measuring the amount of remaining intact DX by gel filtration. Intact DX was detected in fractions 11–15 (Fig. 5b). We confirmed that the amount of sugars in fractions 11–35 corresponded to that of the applied sugars. Therefore, we determined that the intact DX in the hydrolysate remained at 3.3% in fractions 11–15. This means that 96.7% of the DX was hydrolyzed.

**Fig. 6:**
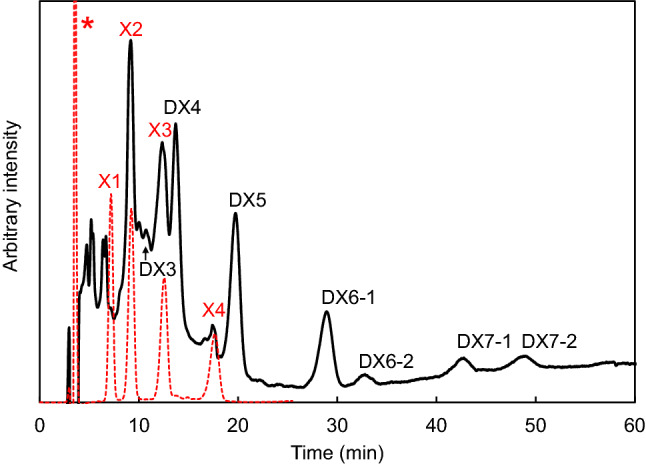
Chromatogram of the hydrolysate from StXyl10 and StXyl11. The hydrolysate was separated by a Sugar-D column (4.6 × 250 mm) with a flow rate of 1.0 mL/min using isocratic elution with 75% acetonitrile and detected with an RI detector. Standards, X1–X4 and DX3 and DX4. The DPs of the sugar peaks eluted later than X4 (DX5–DX7-2) and were estimated by their retention times

The hydrolysis products of the sample were subjected to HPLC. Since the X1 peak in HPLC overlapped with the injection peak, we used a d-xylose analysis kit to quantify X1. The amount of X1 in the sample was 0.94%, meaning that X1 was hardly detectable. The sample contained X2–X4, DX3 and DX4. The peaks that eluted later than X4 would be XOSs containing β(1→3) linkages due to the DX structure and substrate specificities of the xylanases (Biely et al. 1997). We previously reported the structures of DX3 and DX4 (Yamamoto et al. 2019). DX4 showed two structural patterns, i.e., Xyl-β(1→3)-Xyl-β(1→4)-Xyl-β(1→4)-Xyl and Xyl-β(1→4)-Xyl-β(1→3)-Xyl-β(1→4)-Xyl. Therefore, we classified the larger XOSs into two patterns, DX6-1, DX7-1: XOS having a β(1→3) linkage in the middle and DX6-2, DX7-2: XOS having a β(1→3) linkage at the nonreducing terminus. The DP could then be estimated from the retention times (Fig. 6). From these results, the composition of XOSs contained mainly DX4 (21.3%), xylopentaose having one β(1→3) xylosyl linkage (DX5) (18.6%), xylotriose (X3) (18.4%) and X2 (15.5%). Therefore, the product contained a reduced amount of X1 (0.94%). The hydrolysis ratio of DX was 96.7%, and the yield of XOSs was 95.8%. The ratio of XOSs with β(1→3) linkages was 59.7%, resulting in the effective production of unique XOSs.

We previously prepared XOSs from DX using the commercial enzyme hemicellulase amano 90. The product contained 15.0% X1 with a hydrolysis ratio of 82.0% (Yamamoto et al. 2019). The main products of hemicellulase amano 90 were X1 and X2, containing less XOS with a β(1→3) linkage (40%). The preparation method in this study was suitable for producing XOSs with β(1→3) linkages, which increased from 40% to 59.7%.

## Conclusions

Red alga dulse contains xylan with β(1→3)/β(1→4) linkages. Oligosaccharides with different structures show different biological functions. Our previous preparation method contained many X1- and less β(1→3)-linked XOSs. This study showed efficient XOS production by successive hydrolysis using two endoxylanases (0.5 μg/mL StXyl10 for 4 h at 50 °C and then 2.0 μg/mL StXyl11 for 36 h at 60 °C). The composition of the product was as follows: 0.97% X1 and 95.8% XOSs containing 59.7% β(1→3)-linked XOSs. Our study provides useful information for the production of β(1→3)-linked XOSs. On the other hand, we could not clarify the enzyme–structure–functional relationship. To reveal the specificity of these enzymes, we plan to perform mutational analysis.

## Materials and methods

### Materials

Dulse (*Palmaria palmata* in Japan) was harvested at Usujiri, Hokkaido, Japan and stored at − 30 °C until use (Yamamoto et al. 2019; Kobayashi et al. 2020). X1, X2 and X3 were purchased from Wako Pure Chemical Industries (Osaka, Japan). Xylotetraose (X4), xylopentaose (X5), GX from beechwood, xylan and AX from wheat flour, and insoluble xylan were purchased from Megazyme (Bray, Ireland). A Sugar-D column (4.6 × 250 mm) was purchased from Nacalai Tesque (Kyoto, Japan). A Superdex Peptide 10/300 GL column was purchased from GE Healthcare (Tokyo, Japan). Genomic DNA of *S*. *thermogriseus* NBRC 100,772 was obtained from the NITE Biological Resource Center (NBRC). All other reagents were obtained from Wako Pure Chemical Industries Ltd. (Osaka, Japan).

### Preparation of dulse xylan

DX was prepared as previously described (Yamamoto et al. 2019). Specifically, frozen dulse was lyophilized and homogenized into a powder. The powder was delipidated with chloroform–methanol (1:2, v/v) and then dried. DX was extracted from the delipidated powder. The powder was suspended in 40 volumes (v/w) of distilled water and autoclaved at 121 °C for 20 min. The supernatant of the sample was mixed with urea (8 M final concentration), centrifuged and then dialyzed against distilled water using a dialysis tube (molecular weight cut off of approximately 14 kDa; EIDIA Co., Ltd., Tokyo, Japan). The solution was centrifuged at 15,000×*g* for 5 min to remove small amounts of insoluble materials, and the supernatant was lyophilized. Then, the sample was dissolved in water (10 mg/mL) and purified with 80% ethanol precipitation. The dried precipitate was used as DX. The DP of DX was determined by the amount of total sugars and reducing sugars by the phenol-sulfate method (Dubois et al. 1951) and 3,5-dinitrosalicylic acid method (DNS) (Miller 1959), respectively. The purity of the prepared DX was 30.8%, and the average DP of the product was 29.

### Preparation of xylanases

The genes encoding xylanase, StXyl10 (DNA Data Bank of Japan AN: LC603131) and StXyl11 (AN: LC603130), were amplified by polymerase chain reaction (PCR) using the genomic DNA of *S*. *thermogriseus* as a template and the following sets of primers: 5′- ACATATGGCCGAGAGCACACTCGGCGC-3′ (StXyl10-forward) and 5′- TAAGCTTTCAGGTGCGGATCCAGCGCT-3′ (StXyl10-reverse); and 5′-ACATATGGACACCTACGTCGACACGAACCA-3′ (StXyl11-forward) and 5′-TAAGCTTTCAGCTCGTACTGCAGGAGACCG-3′ (StXyl11-reverse), where underlines indicate the restriction enzyme sites. Then, the genes were cloned into the *Nde*I-*Hin*dIII site of pET28a to construct expression vectors of pET28a(StXyl10) and pET28a(StXyl11). The recombinant proteins were expressed in *Escherichia coli* BL21 (DE3) cells (Agilent Technologies, Palo Alto, CA, USA) harboring each expression vector and purified as previously described (Kumagai et al. 2011). The purity of the proteins was confirmed by SDS-PAGE. The protein concentrations were determined by their absorbance at 280 nm using their respective molar extinction coefficients.

### Zymography

StXyl10 without heat treatment was subjected to native-PAGE with a 12.5% acrylamide gel. After electrophoresis, the gel was washed twice with 20 mM sodium phosphate buffer (pH 7.0) for 10 min. To carry out the enzymatic reaction, the gel was placed on an agar plate containing 0.5% GX in 20 mM sodium phosphate buffer for 30 min at 50 °C. The agar gel was stained with 0.3% (w/v) Congo red solution for 10 min at room temperature and destained with 1.0 M NaCl.

### Xylanase activity

Xylanase activity was determined by the DNS method of measuring the amount of reducing sugars released by the reaction of 10 mg/mL BX, 1.0 μg/mL StXyl10 or StXyl11, and 10 mM sodium phosphate buffer (pH 6.5) at 60 °C for 10 min. One unit of xylanase was defined as the amount of enzyme that liberates reducing sugars equivalent to 1.0 μmol X1 per minute. The optimal pH of the xylanases was determined using 10 mM sodium citrate buffer for pH 4.0–6.0, 10 mM sodium phosphate for pH 6.0, 10 mM Tris–HCl buffer for pH 8.0 and 10 mM glycine–NaOH buffer for pH 9.0–10.0 at 60 °C for 10 min. The optimal temperature was determined between 30–80 °C using 10 mM sodium phosphate buffer (pH 6.5) for 10 min. The thermal stability of the xylanases was assessed by heat treatment at 50–70 °C for 0–24 h in 10 mM sodium phosphate buffer (pH 6.5). The remaining activity was measured under standard conditions. The kinetic parameters of the xylanases for xylan were determined by the Michaelis–Menten equation using Origin 6.0 software (OriginLab Corporation, USA). The activity was assayed in 10 mM sodium phosphate buffer (pH 6.5) at 60 °C containing 0.25–20.0 mg/mL GX or DX. All activity assays were performed in triplicate.

### Hydrolysis of xylan

Hydrolysis of xylan (AX, DX and GX) was performed in a reaction mixture containing 10 mM sodium phosphate (pH 6.5) and 10 mg/mL xylan for 24 h at 50 °C for 1.0 μg/mL StXyl10 and at 60 °C for 1.0 μg/mL StXyl11. To determine the hydrolysis conditions of DX, 10 mg/mL DX was hydrolyzed in 10 mM sodium phosphate (pH 6.5) at 50 °C by 0.1–2.0 μg/mL StXyl10 for 4 h and 0.5 μg/mL StXyl10 for 1–12 h. Evaluation of the hydrolysis of DX conditions by StXyl11 was performed in 10 mg/mL DX and 10 mM sodium phosphate (pH 6.5) at 60 °C with 0.5–8.0 μg/mL StXyl11 for 24 h and 2.0 μg/mL StXyl11 for 1–36 h. Evaluation of the successive hydrolysis of DX by StXyl10 and StXyl11 was performed in two steps. First, 10 mg/mL DX was hydrolyzed in 10 mM sodium phosphate (pH 6.5) at 50 °C by 0.5 μg/mL StXyl10 for 4 h. Then, StXyl10 was inactivated by heating at 100 °C for 5 min. The hydrolysate was hydrolyzed at 60 °C by 0.5 or 2.0 μg/mL StXyl11 for 24 or 36 h.

### Thin-layer chromatography

The hydrolysis products of xylan were analyzed by TLC using a silica gel 60 plate (Merck KGaA, Darmstadt, Germany). The products were developed two times with a 2:1:1 (v/v/v) mixture of 1-butanol, acetic acid, and water. The products were detected by spraying a 2:2:100:15 (w/v/v/v) mixture of diphenylamine, aniline, acetone and 80% phosphate, followed by heating at 100 °C for 10 min using a dry heat block. X1–X3, DX3 and DX4 were used as standards. The amount of each product was semiquantitatively evaluated by imaging with ImageJ (Wayne Rasband (NIH), USA). The image of each TLC plate was converted to grayscale and inverted. Then, the amount of each product was evaluated from the density (Hwang et al. 2010).

### Evaluation of hydrolysis products

The distribution of hydrolysis products was analyzed using high-performance liquid chromatography (HPLC) with a Superdex Peptide 10/300 GL column preequilibration with 0.3 M NaCl as previously described (Kumagai et al. 2016). The samples were eluted at 0.5 mL/min and fractionated every 1 min. The amount of sugars was determined by the phenol-sulfate method. The hydrolysis ratio of DX was determined as follows: (the amount of unhydrolyzed DX/the amount of intact DX) × 100 (%).

The distribution of oligosaccharides was analyzed using HPLC equipped with a Sugar-D column (4.6 × 250 mm, Nacalai Tesque, Kyoto, Japan) with a column oven temperature of 40 °C. The products were eluted with an isocratic elution system of acetonitrile/water (4:1, v/v) at a flow rate of 1.0 mL/min, and the products were detected with an RI detector. X1–X3, DX3 and DX4 were used as standards. The amount of X1 was determined using a d-xylose analysis kit (Megazyme, Ireland).

## Data Availability

All data generated or analyzed during this study are included in this published article.

## Abbreviations

AN: Accession number
AX: Arabinoxylan
CBM: Carbohydrate-binding module
DNS: 3,5-Dinitrosalicylic acid
DP: Degree of polymerization
d-Xylose: X1
DX: Dulse xylan
DX3: β(1→3) Xylosyl xylobiose
DX4–DX6: XOSs containing a β(1→3) xylosyl linkage
HPLC: High-performance liquid chromatography
StXyl10: GH10 xylanase from *S*. *thermogriseus*
StXyl11: GH11 xylanase from *S*. *thermogriseus*
GX: Glucuronoxylan
TLC: Thin-layer chromatography
XOSs: Xylooligosaccharides
